# Association between C-reactive protein to albumin ratio and major adverse cardiac events in patients with stable coronary artery disease treated by percutaneous coronary intervention

**DOI:** 10.3389/fcvm.2026.1746075

**Published:** 2026-03-19

**Authors:** Zhiye Zou, Kai Lai, Shuiqing Gui, Xisi He

**Affiliations:** Department of Critical Care Medicine, Shenzhen Second People’s Hospital, The First Affiliated Hospital of Shenzhen University, Shenzhen, China

**Keywords:** C-reactive protein to albumin ratio, major adverse cardiac events, nonlinear relationship, percutaneous coronary intervention, stable coronaryartery disease

## Abstract

**Objective:**

The purpose of this study was to explore the connection between the C-reactive protein to albumin ratio (CAR) and major adverse cardiac events (MACE) in individuals with stable coronary artery disease (CAD) who underwent percutaneous coronary intervention (PCI).

**Methods:**

This study employed a retrospective cohort design and involved 196 individuals who had recently been diagnosed with stable CAD and received elective PCI. Participants were categorized into four groups according to their CAR quartiles, with the lowest quartile (Q1) serving as the reference group. The primary endpoint was MACE, which was defined as a combination of all-cause mortality, non-fatal myocardial infarction, and non-fatal stroke. To evaluate the association between CAR and MACE, multivariable Cox regression, Kaplan–Meier survival analysis, and restricted cubic spline analyses were utilized.

**Results:**

Among the 196 participants (average age, 72.85 ± 10.11 years), 27 (13.78%) experienced MACE during an average follow-up period of 742 days. The rate of MACE rose significantly across the CAR quartiles (Q1: 8.16%, Q2: 6.12%, Q3: 8.16%, Q4: 32.65%; *p* < 0.001). In the multivariable analysis, compared to patients in the lowest CAR quartile (Q1), those in the highest quartile (Q4) had a significantly increased risk of MACE (Model 3: HR = 4.87, 95% CI: 1.40–16.95, *p* = 0.013). A notable nonlinear relationship was observed between CAR and MACE, with an inflection point at 0.030. Analyses of subgroups indicated a persistent association between increased CAR and MACE across all evaluated strata, with no significant interactions found (all P interaction > 0.05).

**Conclusions:**

Among patients with stable CAD who received PCI, a nonlinear association was found between CAR and MACE.

## Introduction

Despite advancements in percutaneous coronary intervention (PCI) and optimal medical therapy, stable coronary artery disease (CAD) remains a leading global cause of illness and death (1, 2). It is vital to accurately stratify risk in order to identify patients who might benefit from more intensive monitoring and targeted preventive measures. While traditional risk factors such as diabetes, hypertension, and dyslipidemia are important, they do not completely encompass the residual inflammatory burden that drives the progression of atherosclerosis and subsequent major adverse cardiac events (MACE) (3–5).

C-reactive protein (CRP) and serum albumin are two easily obtainable laboratory indicators that reflect systemic inflammation and nutritional status, respectively (6, 7). The C-reactive protein-to-albumin ratio (CAR) combines these two physiological processes. It has emerged as a significant prognostic marker in critically ill patients suffering from malignancy, sepsis, or acute coronary syndrome (8–10). Additionally, multiple studies have identified a correlation between elevated CAR levels and the severity of coronary artery lesions (11–13). Nevertheless, the specific relationship between CAR and long-term outcomes in patients with stable CAD who are undergoing elective PCI is still not thoroughly investigated. Consequently, we set out to explore the connection between CAR and MACE in a retrospective cohort of individuals recently diagnosed with stable CAD who were treated via elective PCI.

## Materials and methods

### Data source

The raw data were acquired from the Dryad Digital Repository (https://datadryad.org/stash), specifically from the dataset located at https://doi.org/10.5061/dryad.fn6730j, which was initially published by Suzuki S and colleagues in PLoS One (2019; 14(7): e0219044) (14). In accordance with Dryad's terms of service, the dataset can be freely accessed for secondary analysis, thereby protecting the authors’ rights.

### Study design and population

This study employed a retrospective cohort design to evaluate data from patients diagnosed with stable CAD who underwent elective PCI at a single institution from October 2014 to October 2017. Individuals with a background of previous myocardial infarction, cancer, or incomplete baseline CAR values were excluded from this analysis. Data collection occurred upon the patients’ hospitalization. It encompassed a range of factors, including clinical features, medical history, key CAD risk factors, accompanying medical conditions, laboratory findings, electrocardiograms, echocardiograms, angiographic outcomes, medications prescribed at discharge, and information gathered during post-discharge follow-ups. All data were anonymized entirely before access was granted. The research conformed to the ethical standards established in the Declaration of Helsinki.

### Definitions and endpoints

The primary outcome measured was MACE, defined to include all-cause mortality, non-fatal myocardial infarction, and non-fatal stroke. Participants were followed for a median of 742 days.

### Statistical analysis

In this study, 196 patients were divided into four equal-sized groups based on their baseline CAR quartiles. Continuous data are presented as mean ± standard deviation (SD) or median [interquartile range (IQR)]. These were compared among the groups using either one-way analysis of variance (ANOVA) or the Kruskal–Wallis test, depending on the situation. For categorical data, frequencies and percentages [n (%)] are presented and analyzed using the *χ*^2^ test or Fisher's exact test, as appropriate. The relationship between CAR and MACE was examined through both univariate and multivariate logistic regression analyses. The multivariable models were established as follows: Model 1 remained unadjusted; Model 2 included adjustments for age, gender, hypertension status, history of smoking, diabetes, and body mass index (BMI); and Model 3 included all variables from Model 2 along with estimated glomerular filtration rate (eGFR), total cholesterol (T-chol), low-density lipoprotein cholesterol (LDL-C), and left ventricular ejection fraction (LVEF). To assess possible non-linear associations between CAR and MACE, restricted cubic spline (RCS) analysis was employed. Furthermore, subgroup analyses were conducted to evaluate the robustness of the association across various patient groups. Cumulative incidence was estimated using the Kaplan–Meier method, and comparisons among CAR quartiles were performed using the log-rank test. Patients were censored at the date of last follow-up if no MACE occurred, or at the date of death from non-cardiovascular causes. All statistical computations were carried out using R software (version 4.2.2) and MSTATA software (http://www.mstata.com). A two-sided P-value of less than 0.05 was deemed statistically significant.

## Results

### Baseline characteristics

A total of 196 patients (average age 72.85 ± 10.11 years) participated in the study, including 136 (69.39%) males. Of these participants, 27 (13.78%) experienced MACE during follow-up. The patient population was assessed based on CAR distribution, and the CAR ratios were divided into four quartiles: Q1 (0.089), with 49 patients per group. All pertinent variables are presented in Table 1. The analysis indicated significant discrepancies in various parameters, such as age, left ventricular ejection fraction (LVEF), hemoglobin (Hb), estimated glomerular filtration rate (eGFR), high-density lipoprotein (HDL) cholesterol, and MACE occurrence (*p* < 0.05). Patients with higher CAR ratios tended to exhibit common traits, such as older age and lower LVEF, Hb, eGFR, and HDL levels. Additionally, the occurrence of MACE significantly increased across higher CAR quartiles (*p* < 0.001).

**Table 1:** Baseline characteristics of participants.

Variables	Total (*n* = 196)	Tertials of CAR				P-value
		Q1 (*n* = 49) <0.012	Q2 (*n* = 49) 0.012-0.029	Q3 (*n* = 49) 0.029-0.089	Q4 (*n* = 49) >0.089	
Age, years	72.85 ± 10.11	68.73 ± 9.18	74.57 ± 9.52	72.63 ± 10.48	75.45 ± 10.16	0.004
Male sex, n (%)	136 (69.39)	36 (73.47)	31 (63.27)	35 (71.43)	34 (69.39)	0.718
BMI	23.56 ± 3.89	23.46 ± 3.58	23.46 ± 3.51	24.29 ± 4.87	23.05 ± 3.45	0.462
SBP, mmHg	136.43 ± 20.51	136.69 ± 13.50	136.57 ± 20.06	134.47 ± 23.67	138.00 ± 3.62	0.864
DBP, mmHg	77.49 ± 13.45	79.20 ± 10.56	74.20 ± 12.48	77.24 ± 14.89	79.31 ± 15.11	0.201
Hypertension, n (%)	146 (74.49)	38 (77.55)	35 (71.43)	37 (75.51)	36 (73.47)	0.911
Dyslipidemia, n (%)	96 (48.98)	32 (65.31)	15 (30.61)	27 (55.10)	22 (44.90)	0.005
Diabetes mellitus, n (%)	70 (35.71)	21 (42.86)	17 (34.69)	19 (38.78)	13 (26.53)	0.375
Atrial fibrillation, n (%)	26 (13.27)	5 (10.20)	6 (12.24)	6 (12.24)	9 (18.37)	0.660
OCI, n (%)	33 (16.84)	7 (14.29)	9 (18.37)	6 (12.24)	11 (22.45)	0.542
PAD, n (%)	50 (25.51)	11 (22.45)	12 (24.49)	13 (26.53)	14 (28.57)	0.911
Past smoker, n (%)	97 (49.49)	27 (55.10)	22 (44.90)	26 (53.06)	22 (44.90)	0.638
LVEF, %	63.19 ± 9.83	66.24 ± 6.89	63.45 ± 9.03	62.67 ± 9.48	60.41 ± 12.48	0.031
Hb, g/dL	13.56 ± 2.03	14.27 ± 1.60	13.62 ± 1.98	13.60 ± 2.34	12.76 ± 1.89	0.003
eGFR, mL/min/1.73m^2^	60.82 ± 24.82	69.06 ± 18.44	61.47 ± 28.59	54.08 ± 24.50	58.65 ± 24.98	0.023
AST, U/L	24.67 ± 11.06	23.57 ± 6.16	25.16 ± 12.02	22.73 ± 8.35	27.20 ± 15.25	0.199
ALT, U/L	20.85 ± 12.34	21.24 ± 10.04	20.73 ± 12.95	19.90 ± 10.75	21.51 ± 15.25	0.923
T-chol, mg/dL	185.04 ± 32.85	191.05 ± 33.77	188.84 ± 22.58	182.78 ± 40.51	177.50 ± 1.27	0.164
TG, mg/dL	132.20 ± 86.68	134.01 ± 99.09	130.20 ± 84.88	143.51 ± 74.84	121.07 ± 7.38	0.642
HDL, mg/dL	49.99 ± 13.05	54.14 ± 11.91	53.08 ± 15.08	45.69 ± 11.74	47.04 ± 11.37	0.001
LDL, mg/dL	109.91 ± 27.79	114.45 ± 25.34	107.84 ± 20.45	111.79 ± 33.60	105.55 ± 30.00	0.392
HbA1c, %	7.29 ± 13.29	6.33 ± 0.87	6.46 ± 0.86	6.26 ± 0.87	10.10 ± 26.54	0.405
Multivessel disease, n (%)	7.29 ± 13.29	6.33 ± 0.87	6.46 ± 0.86	6.26 ± 0.87	10.10 ± 26.54	0.962
Bifurcation lesions, n (%)	97 (49.49)	28 (57.14)	22 (44.90)	27 (55.10)	20 (40.82)	0.301
LMT lesions, n (%)	13 (6.63)	1 (2.04)	6 (12.24)	2 (4.08)	4 (8.16)	0.213
Ostial lesions, n (%)	28 (14.29)	6 (12.24)	9 (18.37)	6 (12.24)	7 (14.29)	0.801
Calcified lesions, n (%)	28 (14.29)	2 (4.08)	10 (20.41)	8 (16.33)	8 (16.33)	0.112
CTO lesions, n (%)	12 (6.12)	6 (12.24)	3 (6.12)	0 (0.00)	3 (6.12)	0.079
MACE, n (%)	27 (13.78)	4 (8.16)	3 (6.12)	4 (8.16)	16 (32.65)	<0.001

CAR, C-reactive protein to albumin ratio; BMI, body mass index; SBP, systolic blood pressure; DBP, diastolic blood pressure; eGFR, estimated glomerular filtration rate; OCI, old cerebral infarction; PAD, peripheral artery disease; Hb, hemoglobin; LVEF, left ventricular ejection fraction; AST, aspartate aminotransferase; ALT, alanine aminotransferase; T Chol, total cholesterol; TG, triglyceride; HDL Chol, high density lipoprotein cholesterol; LDL Chol, low density lipoprotein cholesterol; HbA1c, hemoglobin A1c; LMT, left main trunk; CTO, chronic total occlusion; MACE, major adverse cardiac events.

### Multivariate analyses results using the logistic regression model

 Table 2 displays the findings from both unadjusted and adjusted multivariable logistic regression analyses. When using the first quartile of CAR as the reference category, higher CAR levels (treated as a continuous variable) were significantly positively associated with an elevated risk of MACE. Following the adjustment for confounding factors such as age, sex, hypertension, previous smoking history, diabetes mellitus, BMI, eGFR, T-chol, LDL, and LVEF, the highest quartile of CAR (Q4) was strongly linked to an increased likelihood of experiencing MACE (HR: 4.87, 95% CI: 1.40–16.95, *p* = 0.013).

**Table 2:** Association between CAR and MACE in different models.

Variables	Model 1		Model 2		Model 3	
	HR (95%CI)	P	HR (95%CI)	P	HR (95%CI)	P
CAR quantile						
1	1.00 (Reference)		1.00 (Reference)		1.00 (Reference)	
2	0.80 (0.18∼3.59)	0.774	0.82 (0.18∼3.76)	0.800	0.68 (0.14∼3.29)	0.633
3	1.01 (0.25∼4.04)	0.990	1.18 (0.29∼4.91)	0.815	0.99 (0.22∼4.45)	0.991
4	4.72 (1.58∼14.16)	**0** **.** **006**	4.20 (1.28∼13.73)	**0**.**018**	4.87 (1.40∼16.95)	**0**.**013**

HR, Hazard Ratios; CI, Confidence Interval.

Model 1: no adjusted.

Model 2: adjusted for age, male sex, hypertension, past smoker, diabetes mellitus, BMI.

Model 3: adjusted for model 2 plus eGFR, T-chol, LDL, LVEF.

### Kaplan–Meier survival analysis by Car quartiles

**Figure 1:**
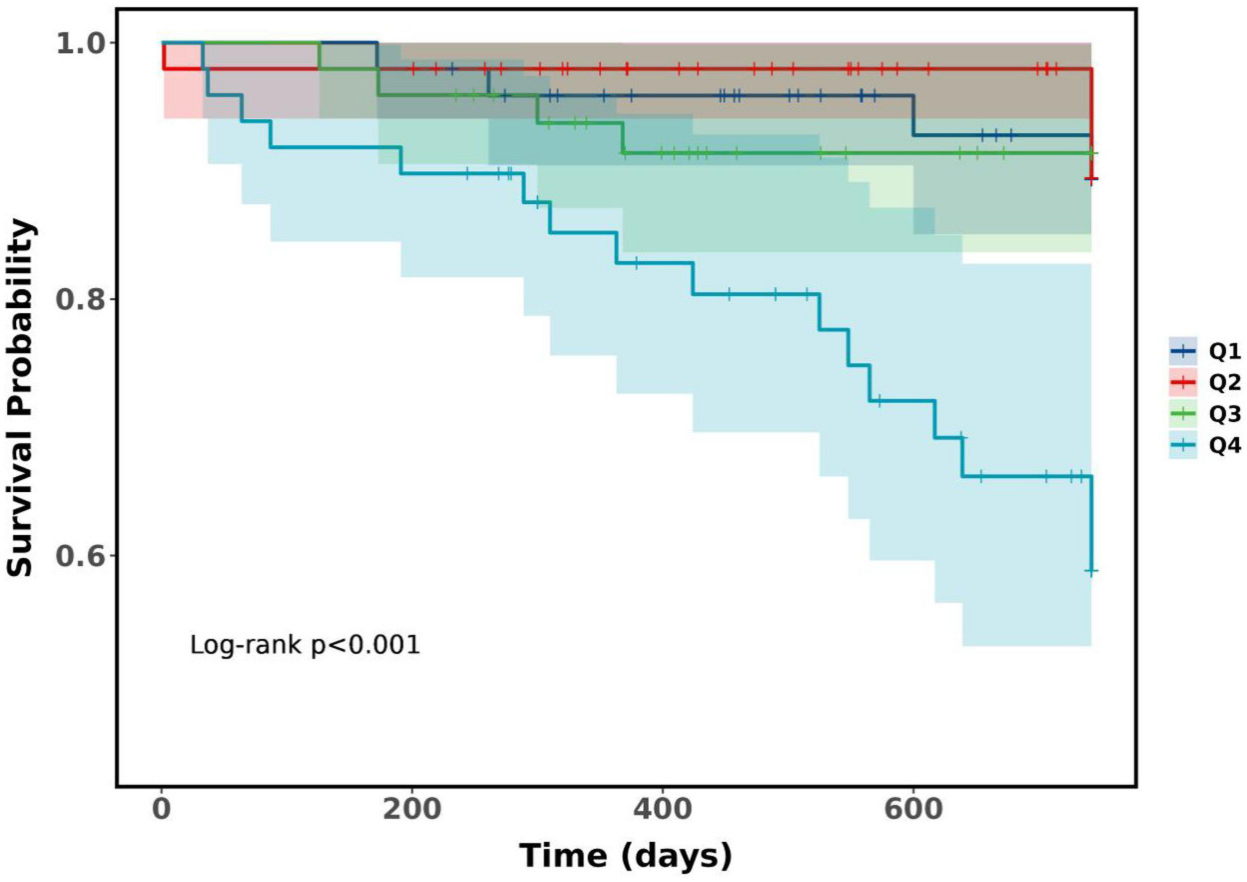
Kaplan–Meier survival analysis curves for MACE.

Figure 1 displays the Kaplan–Meier analysis, which demonstrated that patients in Quartile 4 had significantly lower cumulative survival free from MACE than those in Quartile 1–Quartile 3 (log-rank *p* < 0.001).

### Nonlinear relationship

**Figure 2:**
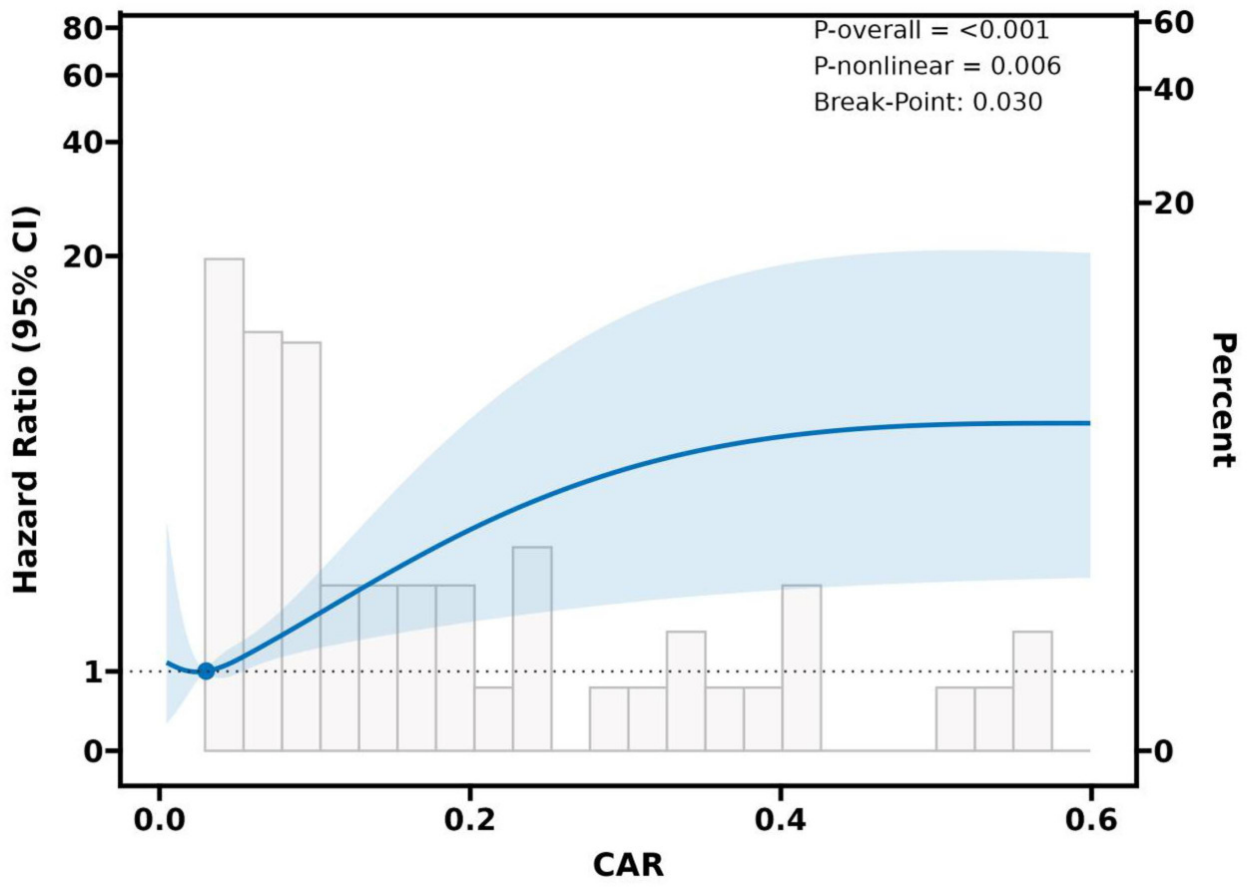
The nonlinear relationship between CAR and MACE.

The relationship between continuous CAR and MACE risk was nonlinear, as demonstrated by the restricted cubic spline analysis (see Figure 2). The curve showed a progressive increase in MACE risk, aligned with increasing CAR levels, with a more pronounced effect observed at higher CAR values. An important upward trend in the adjusted hazard ratio (HR) was observed when the CAR exceeded approximately 0.030. This inflection point indicates a threshold effect, suggesting that the risk of MACE rises significantly beyond this level.

### Subgroup analyses

**Figure 3:**
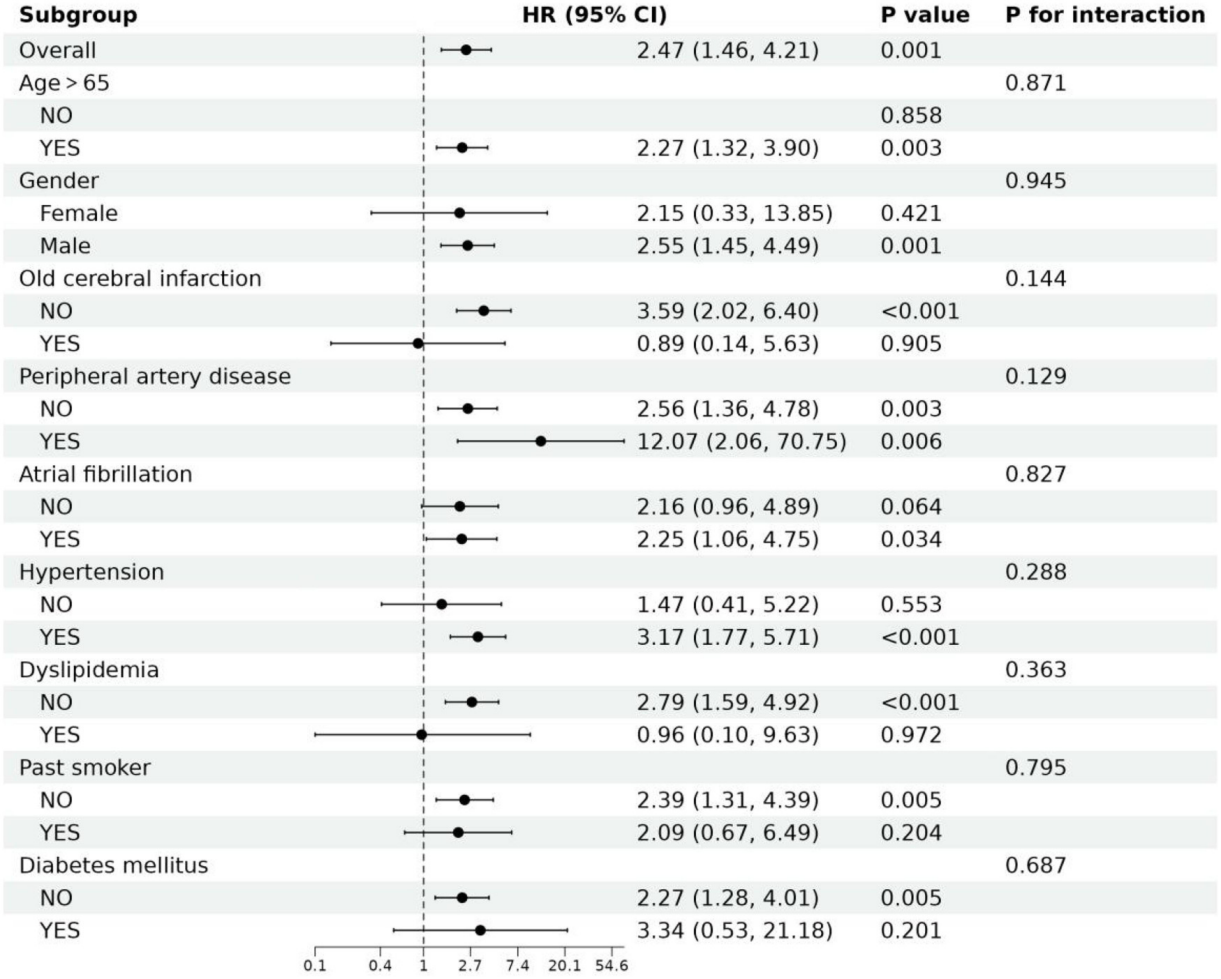
Subgroup analyses of CAR associated with MACE.

As shown in Figure 3, we performed subgroup analyses to assess the relationship between CAR and MACE. The subgroup analyses revealed no notable interactions when accounting for confounding variables, including age (≤65 and >65 years), gender, previous cerebral infarction, peripheral artery disease, atrial fibrillation, hypertension, dyslipidemia, diabetes mellitus, and a history of smoking (all interaction p-values > 0.05).

## Discussion

In a retrospective cohort analysis that included 196 patients diagnosed with stable CAD who underwent elective PCI, we found that CAR was linked to a heightened risk of long-term MACE, independently. Additionally, our analysis revealed a nonlinear association between CAR and MACE, characterized by a notable inflection point at CAR = 0.030, beyond which the risk of MACE significantly escalated. Subgroup analyses showed a consistent association across patient categories, including age, gender, and comorbid conditions, with no notable interactions. These results imply that CAR could be a practical, easily obtainable biomarker for risk assessment in this patient group.

The relationship between the CAR and adverse cardiovascular outcomes can be understood through its reflection of both systemic inflammation and nutritional health. CRP, a marker associated with acute-phase responses, is a recognized indicator of inflammation that contributes to endothelial dysfunction, plaque destabilization, and thrombus formation (6, 15–17). Prior research has highlighted its role in vascular disorders, establishing a link between CRP concentrations and the likelihood of cardiovascular incidents. In a study by Sara JDS et al., it was revealed that coronary endothelial dysfunction (CED) serves as an initial phase of atherosclerosis and correlates with detrimental cardiovascular outcomes. CED has been associated with notably elevated levels of high-sensitivity CRP (hs-CRP), with high-risk hs-CRP independently associated with abnormal coronary vasoreactivity, as reflected in an odds ratio of 1.82 (95% CI 1.25-2.69) (15). A comprehensive review and meta-analysis suggest that heightened hs-CRP levels might worsen the risk linked to lipoprotein(a) [Lp(a)], which contributes to atherogenic pathways and encourages thrombus development (18). Serum albumin not only serves as an indicator of nutritional status but also exhibits anti-inflammatory, antioxidant, and anti-apoptotic properties (7, 19, 20). Numerous studies have shown a significant correlation between low serum albumin levels and several determinants known to increase CAD severity, including elevated blood viscosity, impaired endothelial function, and increased platelet activation (21). Research by Chien SC et al. showed that a reduced serum albumin concentration (<3.5 g/dL) negatively affects outcomes in patients with stable CAD (22). Therefore, combining these two parameters into a single ratio, CAR, may yield a more comprehensive assessment of the underlying inflammatory-nutritional imbalance, which is increasingly recognized as a significant factor driving the progression of atherosclerosis and its subsequent clinical manifestations (5). Our findings align with previous studies, which indicate that higher CAR values are strongly associated with increased rates of reinfarction, heart failure, and mortality (23, 24). For instance, in the context of acute coronary syndromes (ACS), Akboga et al. demonstrated that CAR effectively predicted acute stent thrombosis and a high SYNTAX score (10). Recent evidence underscores the potential of CAR as a marker for CAD and a prognostic indicator in PCI (25–27).

A crucial discovery from our research is the nonlinear relationship between CAR and MACE, with a risk threshold of 0.030. This discovery has significant clinical implications, suggesting that exceeding the CAR threshold may indicate an overburdening of compensatory mechanisms, leading to accelerated vascular damage and a substantial increase in the likelihood of clinical events. Remarkably, this inflection point is below the CAR cut-off values observed in critically ill individuals (28, 29) or in specific cancers (30–32), which may highlight the unique vulnerability of the cardiovascular system to ongoing low-grade inflammation and early nutritional deficits. Nevertheless, given the exploratory nature of this finding and the limited number of events, no clinically actionable threshold can be recommended based solely on this study.

Our study has several limitations that warrant careful consideration. First and foremost, the relatively small sample size and the modest number of MACE events represent a significant limitation. This limited event count, particularly in subgroup analyses, reduces statistical power and may lead to imprecise effect estimates, as reflected in the wide confidence intervals observed. While the association between CAR and MACE remained statistically significant, the breadth of the confidence interval indicates that the actual effect size could be substantially smaller or larger than our point estimate. This imprecision underscores the need for cautious interpretation and highlights the importance of validating these findings in larger, adequately powered prospective cohorts. Second, the laboratory data we used were collected at a single point in time before PCI, limiting our ability to observe dynamic changes in CAR over time. Third, although we accounted for several known confounding factors in our multivariable models, it is essential to acknowledge that other unmeasured or unidentified variables, such as dietary habits, frailty, or unexamined inflammatory markers, may still influence our results. Lastly, due to restrictions in the original dataset, we lacked detailed data regarding specific PCI procedural characteristics, medication adherence during follow-up, and the causes of individual MACE components, which might have offered additional insights into the underlying mechanisms.

## Conclusions

This retrospective cohort analysis involved 196 subjects sourced from the Dryad database. It revealed a nonlinear association between CAR and long-term major adverse cardiovascular events (MACE) in patients with stable coronary artery disease (CAD) undergoing percutaneous coronary intervention (PCI). Below CAR levels of 0.03, the risk of MACE remained relatively constant; however, a notable increase in risk was detected beyond this critical threshold. This benchmark serves as a valuable reference for clinicians when performing risk stratification in this specific patient group. Future investigations should confirm this association through multicenter, prospective trials and examine possible interventions that could address inflammation and nutritional status in patients at high risk, as identified by elevated CAR levels.

## Data Availability

The datasets presented in this study can be found in online repositories. The names of the repository/repositories and accession number(s) can be found below: Dryad (doi:10.5061/dryad.fn6730j).
